# Acetylcholine Acts on Androgen Receptor to Promote the Migration and Invasion but Inhibit the Apoptosis of Human Hepatocarcinoma

**DOI:** 10.1371/journal.pone.0061678

**Published:** 2013-04-19

**Authors:** Huizhen Nie, Qingzhen Cao, Lei Zhu, Yuehua Gong, Jianren Gu, Zuping He

**Affiliations:** 1 State Key Laboratory of Oncogenes and Related Genes, Shanghai Cancer Institute, Renji Hospital, Shanghai Jiao Tong University School of Medicine, Shanghai, China; 2 Stem Cell Research Center, Renji Hospital, Shanghai Jiao Tong University School of Medicine, Shanghai, China; 3 Shanghai Key Laboratory of Reproductive Medicine, Shanghai Jiao Tong University School of Medicine, Shanghai, China; 4 Shanghai Key Laboratory for Assisted Reproduction and Reproductive Genetics, Renji Hospital, Shanghai Jiao Tong University School of Medicine, Shanghai, China; II Università di Napoli, Italy

## Abstract

Hepatocellular carcinoma (HCC) is one of the most fatal cancers. In almost all populations, males have a higher HCC rate than females. Here we sought to explore the roles and mechanisms of acetylcholine (Ach) and androgen receptor (AR) on regulating the fate determinations of HCC. Ach activated AR and promoted its expression in HCC cells. Ach enhanced HCC cell migration and invasion but inhibited their apoptosis. Ach had no obvious effects on the migration, invasion, or apoptosis in AR-negative HCC cells. Elevation of migration and invasion induced by Ach was eliminated in AR-knockdown HCC cells. In contrast, Ach stimulated the migration and invasion but suppressed apoptosis in AR over-expressed HCC cells. Additionally, AR agonist R1881 promoted the migration and invasion but reduced the apoptosis of SNU-449 cells, whereas AR antagonist casodex inhibited the migration and invasion but stimulated the apoptosis of SNU-449 cells. STAT3 and AKT phosphorylation was activated by Ach in HCC cells. Collectively, these data suggest that Ach activates STAT3 and AKT pathways and acts on AR to promote the migration and invasion but inhibit the apoptosis of HCC cells. This study thus provides novel insights into carcinogenesis of liver cancer by local interaction between neurotransmitter Ach and hormone receptor AR in HCC.

## Introduction

Hepatocellular carcinoma (HCC) is among the most lethal cancers, and the survival rate of 5 years for patients with HCC is only 7%. HCC is the 5^th^ most common cancer worldwide and the 3^rd^ most common causes of cancer mortality [1]. In almost all populations, males have a higher HCC rate than females. The male/female ratio of HCC is usually ranging from 2∶1 to 4∶1, and thus androgen has been suggested to regulate the onset and progression of HCC [2]. However, clinical studies using anti-androgen have disappointing results with little beneficial effects of anti-androgen on patients with HCC or even worse survival [3]. The roles of androgen receptor (AR) in HCC remain largely unclear. Study using conditional knockout AR strategy suggests that AR plays dual roles in promoting HCC initiation but suppressing HCC metastasis [3]. Recently, we have demonstrated that AR enhances HCC cell migration and invasion which can be blocked by androgen antagonist casodex (CDX) [4]. AR is a nuclear receptor and regulates gene expression in a variety of tissues during normal development, reproduction, other sexually dimorphic processes, and disease stages including cancers [5], [6]. However, it remains unknown what are the up- and down-regulators for AR in HCC cells.

Neurotransmitters have been confined to the nervous system, and evidence about the presence of neurotransmitters in microorganisms, plants, and lower animals has emerged in recent years. The transmitter acetylcholine (Ach) may function in the regulation of cell fate, such as cellular proliferation, differentiation, and apoptosis. Cholinergic system, including acetylcholinesterase and acetylcholinic receptor, has been detected in HCC, and Ach promotes HCC cell proliferation [7]. Nevertheless, it remains unclear whether Ach plays potential roles in HCC cell migration, invasion, and apoptosis and what are the targets of Ach in regulating the fate of HCC cells.

In this study, we present detailed molecular and cellular evidence supporting that Ach enhances HCC cell migration and invasion but inhibits their apoptosis. Significantly, we have demonstrated that the roles of Ach in regulating HCC cell fate depended on the presence of AR. In addition, phosphorylation of STAT3 and AKT was activated by Ach in HCC cells. Taken together, our data suggest that Ach activates STAT3 and AKT pathways and acts on AR to promote the migration and invasion but inhibit the apoptosis of HCC cells. This study thus provides a new insight into molecular mechanisms in carcinogenesis of liver cancer via the local interaction between neurotransmitter Ach and hormone receptor AR in HCC. Ach and its regulators may be used as novel targets for treating HCC.

## Results

### AChR and AR are Expressed in HCC Cells

To elucidate the relationship between neurotransmitter Ach and endocrine receptor AR in HCC, we first examined AChR mRNA expression in 19 HCC cell lines using real time RT-PCR. AChR include nicotinic acetylcholine receptors (nAChR) and muscarinic acetylcholine receptors (mAChR). Currently there are 12 nAChR subunits (α2–α10 and β2–β4) and 5 mAChR (M1–M5) subtypes [10], [11]. As shown in Fig. S1A, two of the AChR subtypes, namely α7 and M3 AChR, were expressed in 19 HCC cell lines. We further detected AChR and AR protein expression in 7 HCC cell lines. Western blots showed that AChR and AR protein were expressed in these HCC cell lines including SNU-449 cells (Fig. S1B–1D).

### Ach Up-regulates AR Expression in SNU-449 Cells

**Figure 1:**
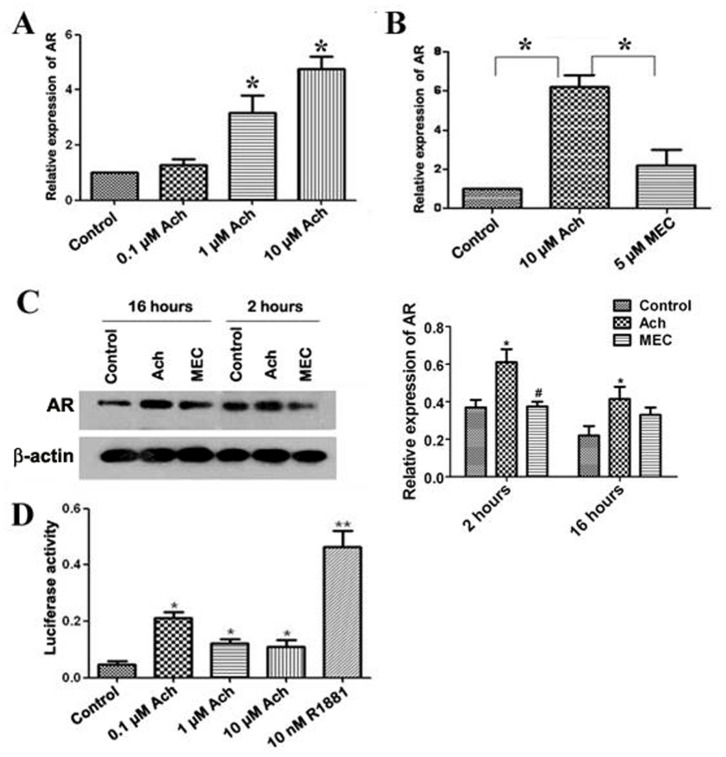
The effects of Ach and mecamylamine (MEC) on AR expression and AR transcriptional activity in SNU-449 cells. (A) mRNA expression of AR was up-regulated by Ach in SNU-449 cells. “*” indicated statistically significant differences (*p*<0.05). (B) mRNA expression of AR was increased by Ach in SNU-449 cells whereas this increase was blocked by MEC. “*” indicated statistically significant differences (*p*<0.05). (C) The protein expression of AR is elevated by Ach and this elevation of AR was blocked by MEC. “*” indicated statistically significant differences (*p*<0.05). (D) Luciferase assay showed AR transcriptional activity by Ach and R1881 in SNU-449 cells using the MMTV-luc reporter plasmid. “*” indicated statistically significant differences (*p*<0.05) while “**” indicated very significant differences (*p*<0.01).

To investigate whether neurotransmitter Ach has effects on the expression of endocrine receptor AR in HCC cells, we treated HCC cell line SNU-449 cells with AChR agonist Ach or AChR antagonist mecamylamine (MEC) and measured mRNA and protein levels of AR. Real time RT-PCR revealed that mRNA expression of AR was up-regulated by Ach in SNU-449 cells in a dose-dependent manner (Fig. 1A). After being treated with Ach (10 µM) for 2 h and 16 h, the protein expression of AR was also elevated (Fig. 1C). In contrast, the enhancement of AR transcription and translation induced by AR was blocked by AChR antagonist MEC (Fig. 1B and 1C). Considered together, these results suggest that Ach up-regulates AR expression in HCC cells.

### Ach Activates AR Transcriptional Activity in SNU-449 Cells

To determine whether Ach activated AR in HCC cells, AR transcriptional activity was measured in SNU-449 cells using a luciferase assay with the MMTV-luc reporter plasmid. Agonist of androgen receptor R1881 was used as a positive control. As expected, AR transcript was enhanced by R1881 in SNU-449 cells. As shown in Fig. 1D, luciferase activity of Ach-treated cells was increased by 2–3 folds compared to the control, implicating that AR is activated by Ach in HCC cells.

### Ach Promotes the Migration and Invasion of HCC Cells via AR

**Figure 2:**
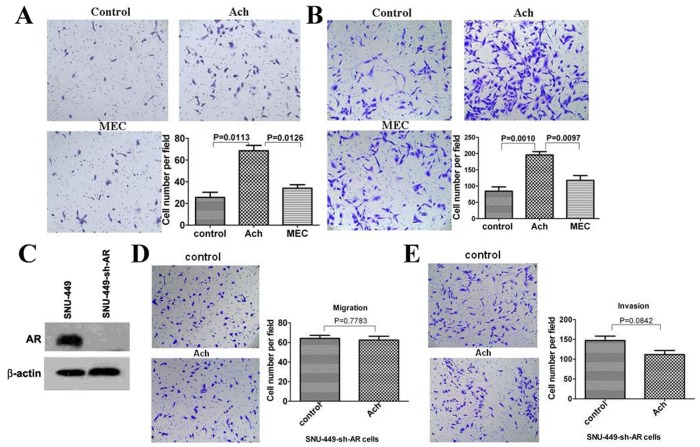
The effects of Ach, MEC, and AR-shRNA on the migration and invasion of SNU-449 cells. (A, B) Transwell cell migration and invasion assays revealed that Ach (10 µM) enhanced the migration (A) and invasion (B) of SNU-449 cells, whereas Ach antagonist MEC (10 µM) blocked this enhancement. “*” indicated statistically significant differences (*p*<0.05). (C) Western blots showed that AR-shRNA knocked down AR expression in SNU-449 cells. SNU-449 cells were infected by the lentivirus vector as a control (sh-control) or the lentivirus expressing shRNA against AR (sh-AR). β-actin served as a loading control of total proteins. (D, E) Transwell cell migration and invasion assays showed that Ach had no effect on migration (D) and invasion (E) in SNU-449-sh-AR cells. SNU-449 cells infected by an empty lentiviral vector (SNU-449-sh-control) served as a control. Quantitative cell numbers were shown.

Using transwell cell migration and invasion assays, we next sought to establish effects of Ach on the migration and invasion in HCC cells. The migration of SNU-449 cells was elevated by Ach compared to the control, whereas the enhancement of Ach-induced migration was blocked by Ach antagonist MEC (Fig. 2A). Similarly, the invasion of SNU-449 cells was enhanced by Ach compared to the control, whereas the increase of Ach-induced invasion was blocked by antagonist MEC (Fig. 2B).

To probe the role of AR in regulation of Ach on cell migration and invasion of HCC cells, a lentiviral system was used to knock down AR via shRNA in SNU-449 cells. Western blots showed that the expression of AR was significantly reduced by shRNA against AR (Fig. 2C). As shown in Fig. 2D and 2E, Ach had no obvious effect in the migration and invasion of SNU-449 cells with AR-knockdown by shRNA compared to the control.

**Figure 3:**
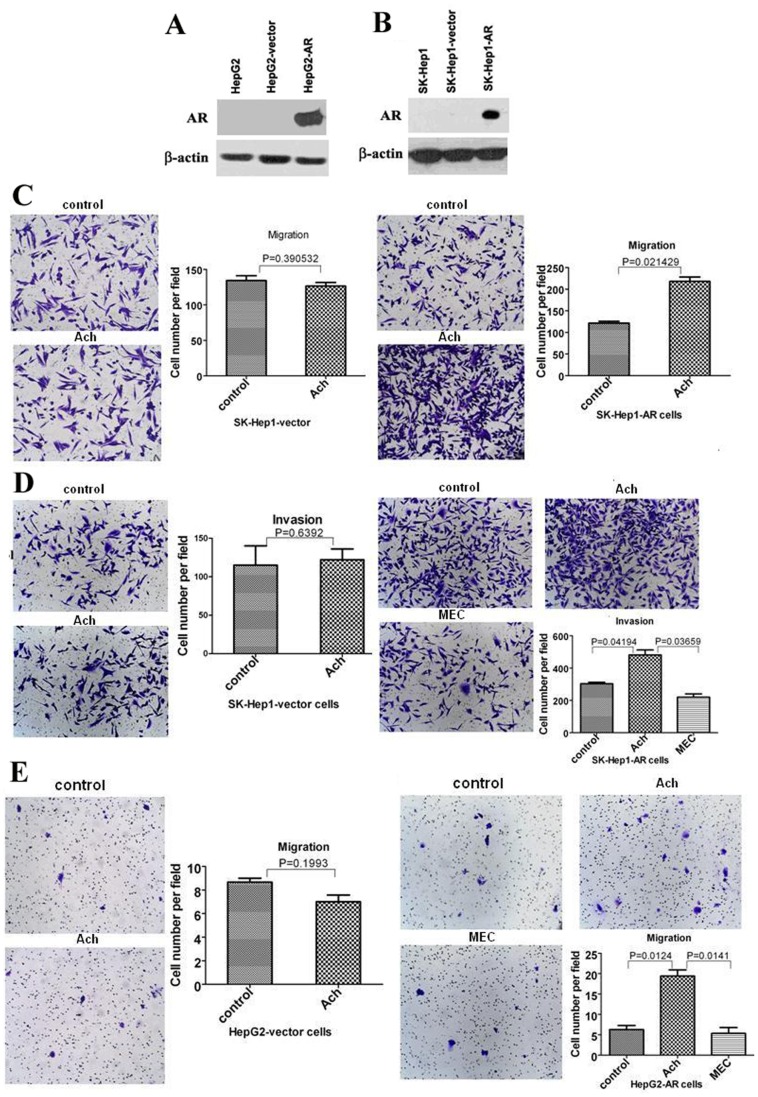
The effects of Ach on migration and invasion of AR negative SK-Hep1 cells and HepG2 cells, SK-Hep1-AR cells, and HepG2-AR cells. (A, B) Western blots revealed expression of AR in HepG2-AR and SK-Hep1-AR cells. HepG2 and SK-Hep1 cells were infected by the lentivirus vector as a control or the lentivirus expressing AR. β-actin served as a loading control of total proteins. (C–D) AR negative SK-Hep1 cells infected with the lentiviral-AR (SK-Hep1-AR) were analyzed for migration (C) and invasion (D) by transwell cell migration and invasion assays after treatment with Ach for 24 h and 48 h respectively. SK-Hep1 cells infected with the lentiviral-empty-vector (SK-Hep1-V) served as a control. (E) AR negative HepG2 cells infected with the lentiviral-AR (HepG2-AR) were analyzed for migration by after treatment with Ach for 24 h. HepG2 cells infected with the lentiviral-empty-vector (HepG2-V) served as a control. “*” indicated statistically significant differences (*p*<0.05).

We further verified the roles of AR in regulation of Ach on the migration and invasion of HCC cells using 2 AR-negative HCC cell lines including HepG2 and SK-Hep1 cells. Western blots displayed that the expression of AR was undetected in HepG2 cells or SK-Hep1 cells (Fig. 3A and 3B). Transwell assays showed that Ach has no effect on the migration of SK-Hep1and HepG2 cells (data not shown). We used a lentiviral overexpression system and AR-negative HepG2 and SK-Hep1 cells to generate AR-positive HepG2 cells and AR-positive SK-Hep1 cells. Western blots revealed that the expression of AR was detected in AR-positive HepG2 cells and AR-positive SK-Hep1 cells (Fig. 3A and 3B). Cell migration and invasion assays revealed that the rates of cell migration and invasion were higher in the Ach-treated AR-positive HepG2 cells and AR-positive SK-Hep1 cells than those in the AR-negative HepG2 cells and SK-Hep1 cells (Fig. 3C–3E). Considered together, these results suggest AR is involved in regulation of Ach on migration and invasion in HCC cells.

### AR Involves in Regulation of Ach on Apoptosis in HCC Cells

**Figure 4:**
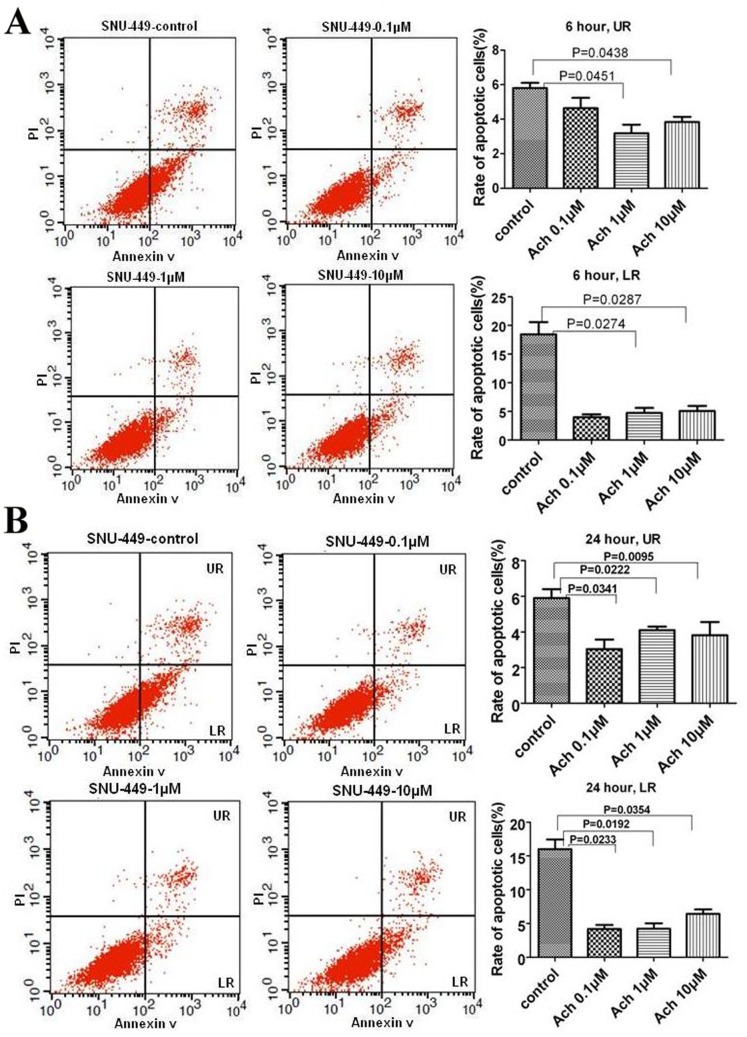
The effects of Ach and MEC on the apoptosis of SNU-449 cells. (A, B) Double staining with Annexin V-FITC and PI showed that early (LR) and late (UR) apoptosis rates of SNU-449 cells were increased by Ach treatment for 6 h and 24 h.

We next determined whether Ach affected HCC cell apoptosis. After being treated with Ach for 6 h or 24 h, both early apoptosis and late apoptosis of SNU-449 cells were diminished (Fig. 4A and 4B). These data imply that Ach inhibits the apoptosis of HCC cells.

**Figure 5:**
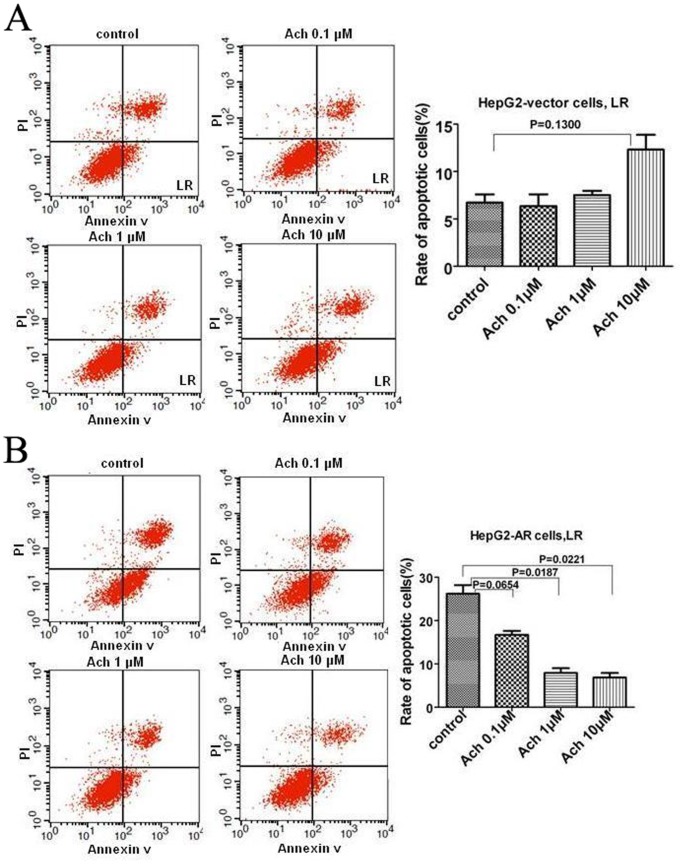
The effects of Ach on apoptosis of AR negative HepG2-V cells and HepG2-AR cells. (A, B) AR negative HepG2 cells infected with the lentiviral-AR (HepG2-AR) were analyzed for apoptosis rates after treatment with Ach for 24 h. HepG2 cells infected with the lentiviral-empty-vector (HepG2-V) served as a control.

To further probe the role of AR in regulation of Ach on apoptosis, we detected the effects of Ach on AR-negative HepG2 cells and AR-positive HepG2 cells. Consistent with the results obtained in transwell assay, 0.1 µM and 1 µM Ach had no effect on early apoptosis in AR-negative HepG2 cells (Fig. 5A), whereas early apoptosis of the cells was increased by 10 µM Ach (Fig. 5A). In contrast, early apoptosis rate in AR-positive HepG2 cells was decreased by Ach in a dose-dependent fashion (Fig. 5B). Collectively, these results reflect that AR is involved in regulation of Ach on early apoptosis in HCC cells.

### The Effects of AR Agonist R1881 and AR Antagonist Casodex (CDX) on the Migration, Invasion, and Apoptosis in HCC Cells

**Figure 6:**
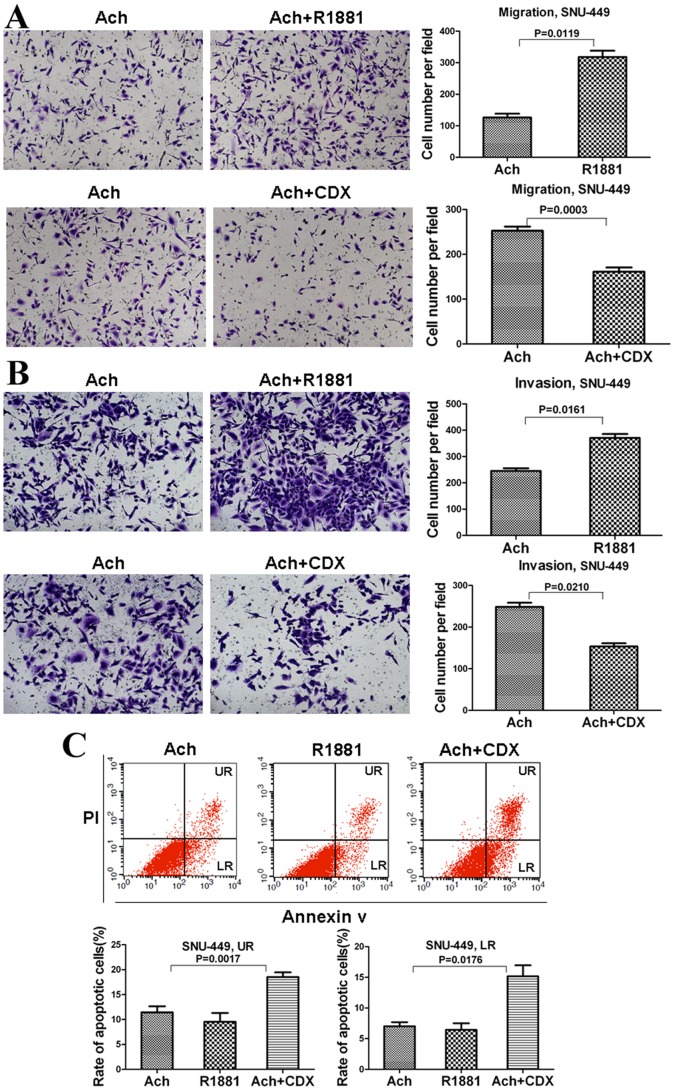
AR agonist R1881 and AR antagonist casodex (CDX) on the migration, invasion, and apoptosis of SNU-449 cells. (A, B) Transwell cell migration and invasion assays showed the effects of R1881 and CDX on the migration (A) and invasion (B) of SNU-449 cells. (C) Double staining with Annexin V-FITC and PI revealed the effects of R1881 and CDX on early (LR) and late (UR) apoptosis rates of SNU-449 cells.

To offer a definite relationship between Ach and AR in regulating the migration, invasion, and apoptosis of HCC cells, we used AR agonist R1881 and AR antagonist casodex (CDX). As shown in Fig. 6A and 6B, R1881 induced more migration and invasion of SNU-449 cells than Ach, whereas CDX blocked the enhancement of migration and invasion by Ach in SNU-449 cells. Conversely, R1881 reduced the early and late apoptosis of SNU-449 cells compared with Ach, whereas CDX coordinated with Ach to increase the early and late apoptosis of SNU-449 cells (Fig. 6C). Collectively, these data further illustrate that Ach acts via AR to control the migration, invasion, and apoptosis of HCC cells.

### Ach Activates STAT3 and AKT Signaling Pathways in SNU-449 Cells

**Figure 7:**
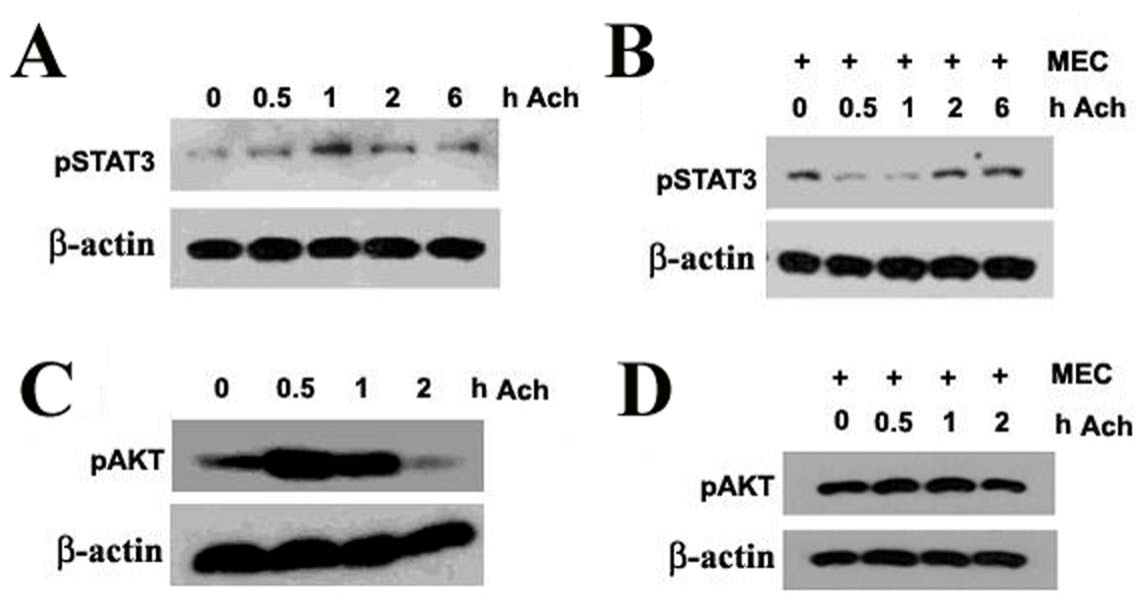
Effects of Ach and MEC on Akt and STAT3 phosphorylation in SNU-449 cells. (A–D) Western blots showed the effects of Ach and MEC on STAT3 phosphorylation (A, B) and Akt phosphorylation (C, D) and in SNU-449 cells. Phosphorylation of STAT3 (A) and Akt (C) was enhanced by Ach in SNU-449 cells, whereas acetylcholine receptor antagonists MEC blocked the effect of Ach on STAT3 (B) and AKT (D) phosphorylation.

To gain novel insights into molecular mechanisms regulating the roles of Ach on HCC cells, we analyzed what signaling pathways were activated. Western blots revealed that STAT3 and AKT phosphorylation of SNU-449 cells was enhanced by Ach (Fig. 7A and 7C). By contrast, STAT3 and AKT phosphorylation induced by Ach was blocked by Ach antagonist MEC (Fig. 7B and 7D). Taken together, these results suggest that Ach activates STAT3 and AKT signaling pathways in HCC cells.

## Discussion

Smoking and environmental factors promote the function of nAChRs on cancers such as lung cancer [12]. In other types of cancers, including pancreas cancer, prostate cancer, breast cancer, and ovarian and nasopharyngeal cancers, nAChRs signaling is also altered [13]. Recently, Ach signaling via autocrine and paracrine system has been reported in HCC cell line and liver cancer tissues [7]. Consistent with this report, herein we found that two of the AChR subtypes, namely α7 and M3, were expressed in 19 HCC cell lines, suggesting that Ach play potential roles in carcinogenesis of liver cancer. It has been identified for the first time the existence of cholinergic system in human hepatocytes and HCC, in which the Ach degradation enzyme, acetylcholine esterase down-regulation in HCC, can increase the Ach level and thereby activate the AChR to promote HCC proliferation and counteract the drug-induced apoptosis [7]. In this study, we have for the first time demonstrated that Ach enhanced cell migration and invasion but suppressed apoptosis in HCC cells. These results suggest that Ach, as a classical transmitter, plays an important role in cancer metastasis and apoptosis of HCC cells.

Sexual hormone receptors, such as AR and estrogen receptor, have been suggested to become therapeutic targets in sexual organ cancers, including prostate cancer and breast cancer [14], [15]. However, their implications in non-sexual organ cancers remain elusive. According to the epidemiological and experimental studies, both androgen and AR could contribute to gender disparity of HCC, but molecular mechanisms of androgen and AR are still largely unknown. It has been reported that higher activity of androgen pathway functions promotes tumor formation in male hepatocarcinogenesis [16]. In hepatocyte AR-knockout mice, AR inhibits HCC metastasis through modulation of cell migration and anoikis [17]. We have previously found that activation of AR promotes HCC cell migration and invasion [4]. In the present study, we demonstrate that AR is present in numerous HCC cell lines, reflecting that AR is involved in HCC. Our results, together with other studies, indicate that AR indeed plays potential roles in HCC metastasis. However, the up- and down-stream targets of AR in regulating HCC cell fate determinations remain largely unknown. Systemic deregulation has been regarded as important basis of cancer onset and development. Endocrine system, nervous system, and immune system interact globally in carcinogenesis. In this study, we sought to explore relationship of neurotransmitters and hormones by investigating effects of Ach on AR mRNA and protein expression in HCC cells. We found that Ach enhances both mRNA and protein expression of AR in SNU-449 cells. In addition, luciferase assay shows that Ach activates AR in SNU-449 cells. Taken together, Ach not only enhances AR expression but activates AR in HCC cells. We also found that AR agonist R1881 promoted the migration and invasion but reduced the apoptosis of SNU-449 cells, whereas AR antagonist casodex (CDX) inhibited the migration and invasion but stimulated the apoptosis of SNU-449 cells. Collectively, these data illustrate that Ach acts via AR to control the migration, invasion, and apoptosis of HCC cells. Notably, using AR knockdown and overexpression in HCC cells, we further demonstrate that AR is essential for Ach to promote the migration and invasion but inhibit the apoptosis of HCC cells.

AR is a ligand-activated transcription factor that mediates the biological responses of androgen. Nevertheless, non-androgenic pathways have also been shown to activate the AR. It has been reported that AR can be activated by other molecules such as IL-6 and HER2/Neu signals [18], [19]. Two signaling transduction pathways, namely PI3K/AKT and JAK/STAT3 pathways, have been shown to activate AR in prostate cancer cells [20]–[22]. Activation of STAT3 and ERK1/2 by nicotine modulates cell proliferation in human bladder cancer cells [23]. In this study, we found that phosphorylation of AKT and STAT3 was elevated by Ach, implicating that Ach activates AKT and STAT3 signaling pathways in HCC cells. Moreover, we have previously demonstrated the existence of a non-neuronal cholinergic autocrine/paracrine system in normal human hepatocytes and its dysregulation in HCC [7]. The present findings further illustrate that the neurotransmitter Ach can regulate the expression of AR. Thus, our studies implicate that there may exist a systemic regulatory system with neural/non-neural neurotransmitters, endocrine, and immune systems as the core elements, and its dysregulation might be critical in hepatocarcinogenesis and HCC progression.

In summary, we have for the first time demonstrated that Ach activates STAT3 and AKT pathways and it acts on AR to promote the migration and invasion but inhibit the apoptosis of SNU-449 cells. This study thus offers a novel concept of locally systemic regulation in carcinogenesis by our novel findings showing the regulation between neurotransmitter Ach and hormone AR in HCC cells. We have also identified Ach and its regulators, including AR, STAT3 and AKT pathways, which could be used as potential targets for the treatment of HCC.

## Materials and Methods

### Cell Culture

All HCC cell lines, including SNU-449 cells, HepG2 and SK-Hep-1 cells, were purchased from ATCC (Manassas, VA,USA). SNU-449 cells were cultured in RPMI-1640 medium (Cat#C11875, Invitrogen) supplemented with 10% fetal bovine serum (FBS, Cat#10099-141, Invitrogen, CA), while HepG2 and SK-Hep-1 cell lines were cultivated in Dulbecco’s modified Eagle medium (Cat#C11965, Invitrogen) supplemented 10% FBS (Invitrogen). All cell cultures were maintained at 37°C in 5% CO_2_ incubator. Ach and mecamylamine (MEC) were purchased from Sigma.

### Lentiviral Overexpression and Transfection

The lentiviral overexpression systems were purchased from System Biosciences (SBI, Mountain View, CA, USA). The overexpression vector was co-transfected with packaging vectors psPAX and pMD2.G at a ratio of 3∶2:1 into the 293T cells [4] using Lipofectamine 2000 (Invitrogen) for 3 days before the virus- containing media were harvested, and 5 mg/ml puromycin (Sigma) was added to the cells for selecting the positive cells 2 days later.

### RNA Extraction and Real-time Reverse Transcription-polymerase Chain Reaction (RT-PCR)

Total RNA was isolated from the cells using TRIzol (Cat#15596, Invitrogen) according to the procedure we previously described [8]. Reverse transcription (RT) was performed using a PrimeScript reverse transcription kit (Cat# DRR037A, TaKaRa) in a reverse transcription reaction at 37°C for 15 min. Real-time PCR was carried out using the SYBR real-time PCR kit (Cat# DRR041A, TaKaRa). The ABI 7300 model real-time PCR system was used for detection (ABI, USA). The primers of chosen genes were as follows: M3 AChR-sense: GGCCTGTGCCGATCTGATTAT, M3 AChR-anti-sense: CGGCCTCGTGATGGAAAAG; α7 AChR-sense: GATGAGCACCTCCTGCACGG, α7 AChR-anti-sense: GATGCCGATGGTGCAGATG; 18S-sense: GCAATTATTCCCCATG, 18S-anti-sense: GGGACTTAATCAACGCAAGC.

### Western Blots

Cells were lysed with RIPA buffer (Santa Cruz) for 30 min on ice. Cell lysates were cleared by centrifugation at 12,000 g, and the concentration of proteins was measured by BCA kit. Western blots were carried out according to the procedure we previously described [9]. After blocking for 1 h at room temperature, the blots were probed respectively with primary antibodies for 3 h and with secondary antibody conjugated with HPR (1∶5,000; Proteintech Group Inc. USA) for 1 h at room temperature. Finally, these membranes were detected by the ECL Plus reagents and visualized by enhanced chemiluminescence (Thermo, USA). Primary antibodies used here included rabbit monoclonal anti-AR (D6F11) (3H8; Cell Signaling Technology, Danvers, MA), rabbit monoclonal anti-nicotinic acetylcholine receptor alpha 7 (ab10096, abcam, USA), rabbit monoclonal anti-pSTAT3, anti-pAKT (195-14; BioCheck, Foster City, CA), and monoclonal anti-β-actin (Sigma).

### Luciferase Assay

SNU-449 cells were seeded at a density of 1×10^5^ cells/well into 24-well culture plates in RPMI 1640 medium containing 10% FBS without antibiotics. The cells were co-transfected with 0.76 µg of the androgen-responsive MMTV-luc firefly luciferase reporter plasmid and 0.04 µg of renilla luciferase plasmid phRL-TK using Lipofectamine 2000 (Invitrogen) according to the manufacturer’s instructions. Cells were treated with Ach for 24 h, and cell lysates were prepared. Luciferase activities were measured using the 120 dual-luciferase reporter assay system (Promega, USA). Firefly luciferase activity was normalized to the activity of renilla luciferase.

### Cell Migration and Invasion Assays

Cell migration assays were carried out according to the manufacturer’s protocols. Cells were cultured with serum-free media in transwell chambers (BD Falcon, USA), and Ach, mecamylamine (MEC), 10^−8^ M R1881 (Sigma), or Ach and 10^−5^ M casodex (CDX) (Sigma) were added to media. The cells were cultured for 24 or 48 h followed by extensive phosphate-buffered saline (PBS) washes. These cells were fixed with 4% PFA and stained with 0.1% crystal violet. The un-migrated cells were removed using cotton swabs, whereas the migrated cells were counted from five random fields under a microscope. For invasion assays, transwell chambers were covered with matrigel (BD Falcon, USA) before a procedure similar to that for the migration assays was performed.

### Cell Apoptosis Assays

Double staining with Annexin V-FITC and PI (BD, USA) was performed for quantification of apoptosis of SNU-449 cells, HepG2-vector cells, and HepG2-AR cells. Both attached cells and the cells in the supernatant were collected, washed twice with ice-cold PBS, and resuspended in 400 µL of binding buffer. Annexin V-FITC and PI were added to the cells and incubated for 15 min in the dark at 4°C. After incubation, the samples were analyzed by a flow cytometry (BD FACSCalibur, USA), and 2.0×10^4^ events per sample were counted.

### Statistical Analysis

All experiments were performed independently at least 3 times. All the values were presented as mean ± SEM. Statistical analysis was performed using a two-tailed Student’s t test, and *p*<0.05 was considered statistically significant.

## Supporting Information

Figure S1 **The expression of AChR and AR in HCC cell lines and normal liver cells.** (A) Real-time RT-PCR showed mRNA levels of α7 AChR and M3 AChR in 19 HCC cell lines and the normal liver cell line THLE-2 cells. Data values were normalized to 18S RNA. (B–D) Western blots revealed protein levels of α7 AChR and AR in various HCC cell lines. β-actin served as a loading control of total proteins.
(TIF)
