# Supplementary figures and images for: Acetylcholine Acts on Androgen Receptor to Promote the Migration and Invasion but Inhibit the Apoptosis of Human Hepatocarcinoma
Source: PLoS One. 2013 Apr 19;8(4):e61678. doi: 10.1371/journal.pone.0061678 (PMC3631145; doi:10.1371/journal.pone.0061678)

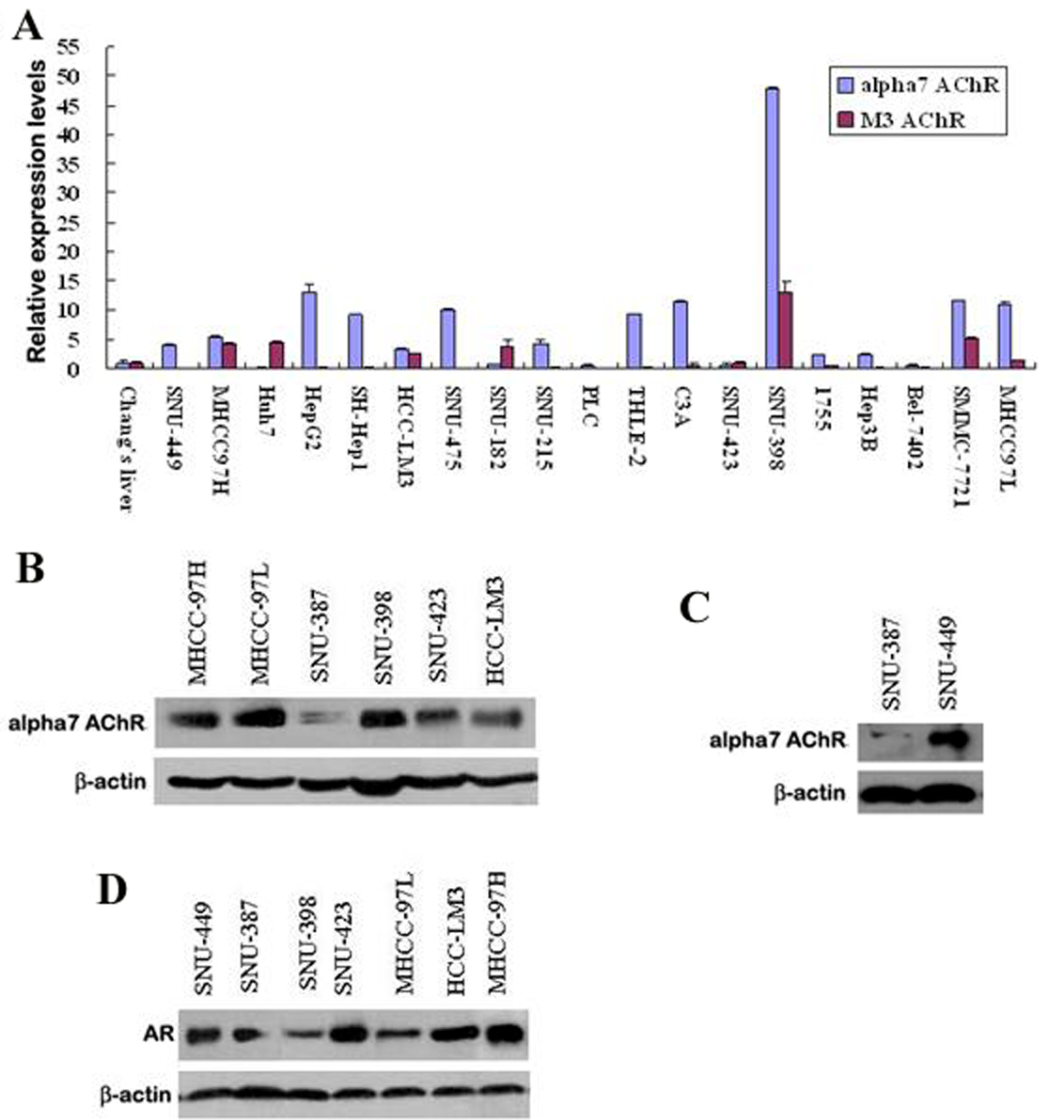

Supplement: Figure S1 — The expression of AChR and AR in HCC cell lines and normal liver cells. (A) Real-time RT-PCR showed mRNA levels of α7 AChR and M3 AChR in 19 HCC cell lines and the normal liver cell line THLE-2 cells. Data values were normalized to 18S RNA. (B–D) Western blots revealed protein levels of α7 AChR and AR in various HCC cell lines. β-actin served as a loading control of total proteins. (TIF) [file pone.0061678.s001.tif]
